# Synergistic Effects of SDS and H_2_O_2_ Combinations on Tracheal Scaffold Development: An In Vitro Study Using Goat Trachea

**DOI:** 10.1155/2024/6635565

**Published:** 2024-01-02

**Authors:** Dhihintia Jiwangga, Ferdiansyah Mahyudin, Gondo Mastutik, Estya Nadya Meitavany, Juliana, Priangga Adi Wiratama

**Affiliations:** ^1^Doctoral Program of Medical Science, Faculty of Medicine, Universitas Airlangga, Surabaya, Indonesia; ^2^Department of Orthopaedic and Traumatology, Faculty of Medicine, Universitas Airlangga, Dr. Soetomo General Academic Hospital, Surabaya, Indonesia; ^3^Department of Anatomic Pathology, Faculty of Medicine, Universitas Airlangga, Surabaya, Indonesia; ^4^School of Biomedical Engineering and Imaging Sciences, King's College London, London, UK; ^5^Faculty of Medicine, Universitas Airlangga, Surabaya, Indonesia

## Abstract

Currently, a tissue-engineered trachea has been popularly used as a biological graft for tracheal replacement in severe respiratory diseases. In the development of tissue-engineered tracheal scaffolds, in vitro studies play a crucial role in allowing researchers to evaluate the efficacy and safety of scaffold designs and fabrication techniques before progressing to in vivo or clinical trials. This research involved the decellularization of goat trachea using SDS, H_2_O_2_, and their combinations. Various quantitative and qualitative assessments were performed, including histological analysis, immunohistochemistry, and biomechanical testing. Hematoxylin and eosin staining evaluated the cellular content, while safranin O-fast green and Masson's trichrome staining assessed glycosaminoglycan content and collagen distribution, respectively. The immunohistochemical analysis focused on detecting MHC-1 antigen presence. Tensile strength measurements were conducted to evaluate the biomechanical properties of the decellularized scaffolds. The results demonstrated that the combination of SDS and H_2_O_2_ for goat tracheal decellularization yielded scaffolds with minimal cellular remnants, low toxicity, preserved ECM, and high tensile strength and elasticity. This method holds promise for developing functional tracheal scaffolds to address severe respiratory diseases effectively.

## 1. Introduction

Respiratory diseases pose a significant burden on global health, affecting millions of people worldwide [1, 2]. Among these conditions, tracheal diseases hold particular importance due to the critical role of the trachea in facilitating proper airflow and respiratory function. Tracheal diseases encompass a range of conditions, including tracheal stenosis, tracheomalacia, and acquired tracheal defects. These diseases can severely impact breathing and quality of life and even lead to life-threatening complications. Therefore, developing effective treatments for tracheal diseases is of utmost importance to improve patient outcomes and enhance global respiratory health.

Conventional treatment methods for tracheal diseases include surgical and pharmacological approaches. Surgical procedures are one of the main interventions in the management of tracheal disease, but they often involve complex and invasive techniques. The variability of the success rates is contingent upon various factors, including the extent and nature of the tracheal condition [3, 4]. Pharmacological interventions, on the other hand, may provide symptomatic relief but fail to address the underlying structural abnormalities of the trachea [5, 6]. These challenges necessitate the exploration of innovative approaches that can overcome the limitations of existing treatments and offer improved solutions for patients with tracheal diseases.

Clinical repair of tracheal injuries, particularly those involving long segment tracheal defects, is challenging. Tracheal defects stem from various causes such as severe trauma, infections, tumor growth, softening, and congenital tracheal stenosis. Clinicians recommend tracheal replacement in cases where the defect length or resection exceeds 50% in adults or 30% in infants. An ideal tracheal substitute should be flexible, biomechanically appropriate, biocompatible, and have low toxicity and immunogenic effects [7–9]. Moreover, the substitute's revascularization capacity needs to support tissue regeneration while inhibiting the development of tissue necrosis or infection [10]. These complex requirements have made it difficult for researchers to develop an ideal tracheal substitute, which currently remains elusive [11].

Tissue engineering has emerged as a promising solution for tracheal disease treatment. By combining principles of biology, engineering, and material science, tissue engineering aims to create functional, living tissue substitutes that can restore proper respiratory function [12]. Tissue-engineered tracheal scaffolds have shown great potential in providing structural support, promoting tissue regeneration, and facilitating the integration of host cells. These scaffolds offer a personalized and regenerative approach to tracheal disease treatment, addressing the specific needs of individual patients and potentially reducing the risk of complications.

Additionally, the process of decellularization has become a widely used technique in the preparation of tissue-engineered trachea (TET). Functionally, this method can eliminate cellular components entirely and enhance the preservation of the extracellular matrix (ECM) while maintaining the three-dimensional structure of the trachea [13, 14]. However, the clinical utilization of tissue-engineered trachea has been constrained by several limitations [7, 15]. For example, an extended preparation period, spanning from several days to several months, is not favorable for patients with acute tracheal defects [16]. Additionally, restricted revascularization is a crucial factor that markedly influences tracheal repair [17].

In the development of tissue-engineered tracheal scaffolds, in vitro studies play a crucial role in allowing researchers to evaluate the efficacy and safety of scaffold designs and fabrication techniques before progressing to in vivo or clinical trials. In vitro models provide a controlled environment for investigating scaffold properties, optimizing their physical and mechanical characteristics, and assessing their biocompatibility. Thus, the objective of this research article is to present an in vitro study aimed at developing and evaluating a novel tracheal scaffold for the treatment of tracheal diseases. The study focuses on the investigation of the combined use of sodium dodecyl sulphate (SDS) and hydrogen peroxide (H_2_O_2_) in scaffold development using goat trachea as a model. The outcomes of this study have the potential to advance tracheal disease treatment strategies, improve patient outcomes, and contribute to the growing field of tissue engineering in respiratory medicine.

## 2. Materials and Methods

### 2.1. Study Design, Sampling, and Population

The study design was a blinded experimental in vitro study using goat tracheas obtained from an abattoir, which were further classified into five groups. The trachea in the first four groups was decellularized using different agents (SDS 0.5%, SDS 1%, H_2_O_2_ 3%, and a combination of SDS 1% + H_2_O_2_ 3%), while the fifth group, the control group, consists of tracheas that were not decellularized. This study uses a simple random sampling, where the size of the sample was calculated using a resource equation, with a minimum sample of three and a maximum of five samples. All goat tracheas with deformities were excluded from the group. This study has been approved by the Animal Care and Use Committee (ACUC) of the Faculty of Veterinary Medicine, Universitas Airlangga, following the ethical guidelines for animal research.

The sample size of the study is determined using the “resource equation” approach based on the literature by Arifin and Zahiruddin, 2017. The formula for the sample size is presented in Table 1.

In this study, the group comparison formula without repetition is used, so the selected formula is

(1) $$\begin{array}{c} \text{Minimum }n=\frac{10}{k}+1,\\ \text{Maximum }n=\frac{20}{k}+1. \end{array}$$

In this study, 5 treatment groups are used, so the sample size in each group is

Minimum sample size per group: 10/5 + 1 = 2 + 1 = 3.

Maximum sample size per group: 20/5 + 1 = 4 + 1 = 5.

So, the sample size per treatment group is a minimum of 3 and a maximum of 5. In this study, the number of samples for each group is determined to be 5 goat tracheas.

### 2.2. Research Material

The protocol for the preparation of research materials was adapted from studies by Go et al. and Batioglu-Karaaltin et al. [15, 18]. The trachea of Capra hircus L. species goats was obtained from freshly slaughtered animals at the abattoir. A 6 cm segment of the trachea was isolated from surrounding connective tissue and placed in a phosphate-buffered saline (PBS) solution containing penicillin/streptomycin/gentamycin/fungizone and then transported to the stem cell unit laboratory at Dr. Soetomo General Academic Hospital. The trachea stored, which was kept at 2°C to 8°C, underwent a washing process using a 1% povidone-iodine solution in PBS for 5 minutes. After that, it was washed again with a 1% penicillin/gentamycin/fungizone/streptomycin solution in PBS to eliminate any residual povidone-iodine. The aforementioned procedure was performed twice. Following the repetition, the 25 trachea samples that underwent washing were randomly divided into five groups for decellularization. Subsequently, all the samples were frozen at −80°C for a duration of 4 hours. Afterward, the temperature of the lyophilized chamber was reduced to −50°C, and the vacuuming process commenced, lasting for a period of 24 hours.

### 2.3. Tracheal Harvesting and Decellularization Procedure

#### 2.3.1. Trachea Sampling Procedure

The goat trachea was obtained from the Pegirian Surabaya Slaughterhouse, with a length of 15–19 cm, consisting of 13 pieces for pre-experimental research and 25 pieces for the main research. The trachea was immediately immersed in ice after being cut and then transported to the Tissue Bank of Dr. Soetomo General Academic Hospital Surabaya.

#### 2.3.2. Decellularization Procedure

This research was preceded by a pre-experimental study consisting of a control group and four types of treatment groups adapted from the study by Batioglu-Karaaltin et al. and the study by Burk et al., with the following details [15, 19]:

*(1) Control Treatment Group*. Control: trachea without treatment.

*(2) Treatment Group A*. Decellularization group using the trachea immersion method in 0.5% SDS solution at 20–22°C temperature. The SDS solution was replaced every 24 hours for the first two days and then every 48 hours for two weeks. Then, the specimens were stored for 24 hours in a freezer set at a temperature of −80°C. Subsequently, the trachea specimens were freeze-dried using a lyophilizer machine for 48 hours.

*(3) Treatment Group B*. Decellularization group using the trachea immersion method in 1% SDS solution at 20–22°C temperature. The SDS solution was replaced every 24 hours for the first two days and then every 48 hours for two weeks. Then, the specimens were stored for 24 hours in a freezer set at a temperature of −80°C. Subsequently, the trachea specimens were freeze-dried using a lyophilizer machine for 48 hours.

*(4) Treatment Group C*. The decellularization group used the trachea immersion method in a combination of 0.5% SDS solution and 3% H_2_O_2_ solution at 20–22°C temperature. The SDS and H_2_O_2_ solutions were replaced every 24 hours for the first two days and then every 48 hours for two weeks. Then, the specimens were stored for 24 hours in a freezer set at a temperature of −80°C. Subsequently, the trachea specimens were freeze-dried using a lyophilizer machine for 48 hours.

*(5) Treatment Group D*. The decellularization group used the trachea immersion method in 3% H_2_O_2_ solution at 20–22°C temperature. The H_2_O_2_ solution was replaced every 24 hours for the first two days and then every 48 hours for two weeks. Then, the specimens were stored for 24 hours in a freezer set at a temperature of −80°C. Subsequently, the trachea specimens were freeze-dried using a lyophilizer machine for 48 hours.

Each treatment group in the pre-experimental study consisted of 1 sample. Each treatment group was histopathologically evaluated using HE staining, compared to the control group, and the three treatment groups with the most acellular tissue were selected.

### 2.4. Sterilization Procedure

The trachea scaffold was packaged in double-layered polyethylene plastic using vacuum sealing and then sent to BATAN (National Nuclear Energy Agency). The trachea scaffold underwent sterilization using radiation at a dose of 25 kGy.

### 2.5. Histological Analysis

The histological analysis was performed in Stem Cell Unit Laboratory, Dr. Soetomo General Hospital, Diagnostic Center Building, 4th Floor, Surabaya. The postdecellularized trachea samples, measuring 1 cm × 1 cm, were subjected to paraffin blocking and placed in a 10% formaldehyde solution. The dehydration process involved immersing the specimens in 70% alcohol for 2 hours, 80% alcohol for 2 hours, 95% alcohol for 2 hours, 96% alcohol for 2 hours (repeated twice), and 96% alcohol for 2 hours. The clearing process was performed by immersing the specimens in xylene for 1 hour, xylene for 1 hour, and xylene for 2 hours. The impregnation process was carried out by placing the specimens in solid 60°C paraffin for 2 hours, followed by another 2 hours in solid 60°C paraffin. The embedding process involved preparing a base mold at 60°C and a cassette at 60°C. The paraffin dispenser valve was pressed onto the base mold until an adequate volume was reached. The specimens were then placed on the bottom of the filled base mold using forceps. The cassette was placed on top of the base mold containing the tissue/specimens. The filled base mold was placed on a cold plate. After waiting for 2–4 minutes, the base mold solidified. The cassette was then removed from the base mold. The paraffin block was ready for cutting/sectioning. The specimens were subsequently stained with hematoxylin and eosin (H&E), safranin O-fast green (both for GAG staining), and Masson's trichrome (collagen) and were analyzed under a microscope for morphology and ECM matrix observation.

For immunohistochemistry analysis, the process began with deparaffinization using xylene 1 for 5 minutes, xylene 2 for 5 minutes, and xylene 3 for 5 minutes. The specimens were then rehydrated with absolute alcohol for 4 minutes, 96% alcohol for 4 minutes, 80% alcohol for 4 minutes, and 70% alcohol for 4 minutes. They were subsequently rinsed with running water for 5 minutes. Endogenous peroxidase was inhibited with 0.5% hydrogen peroxide for 30 minutes. The specimens underwent thorough rinsing using running water for a duration of 5 minutes. Decloaking chamber (at 110°C for 30 minutes) with diva solution was used and then cooled for 30 minutes. The specimens were rinsed with PBS pH 7.4 for 3 minutes. Blocking was performed with 5% normal horse serum for 30 minutes. The primary antibody was applied for 60 minutes. The specimens were rinsed with PBS pH 7.4 for 3 minutes. Universal Link was applied for 30 minutes. The specimens were rinsed with PBS pH 7.4 for 3 minutes. Trekavidin HRP Label was applied for 30 minutes. The specimens were rinsed with PBS pH 7.4 for 3 minutes. DAB + substrate buffer was applied for 2–5 minutes. The specimens were rinsed with running water for 5 minutes. Counterstaining was performed with hematoxylin for 5–10 minutes. The specimens were rinsed with running water for 5 minutes. Dehydration was carried out with 80% alcohol for 5 minutes, 96% alcohol for 5 minutes, and absolute alcohol for 5 minutes. The clearing was performed with xylene 1 for 5 minutes, xylene 2 for 5 minutes, and xylene 3 for 5 minutes. Finally, the specimens were mounted with a cover glass. The markers used were anti-MHC-1.

### 2.6. Tensile Strength Test Procedure

This protocol was adapted from the study by Batioglu-Karaaltin et al. as follows [15, 20]: trachea segments from each treatment group and fresh trachea from experimental animals were taken on the testing day as controls. The trachea was then vertically cut into two parts and positioned on a device holder with a special soft tissue hook. The specific biomechanical measurement device used was the Mini Bionix II universal test device, and the tensile load was applied using an ESIT Load Cell (SPA-10 kg, output: 2.0 mV/V). The specimens were then stretched at a pulling speed of 15 mm/minute, with a sampling rate of 10 Hz. Time, uniaxial width increment, and uniaxial force were measured until the tissue became damaged. Elasticity was calculated for each sample using the load and stress at the point of tissue damage observed in this curve. The variables assessed in this test were rupture force (N), stress (MPa), and elasticity module-rigidity (N/mm).

### 2.7. Cytotoxicity Procedure

The MTT assay is employed to assess the metabolic activity of cells, serving as a marker for cell viability, proliferation, and cytotoxicity. To begin the MTT assay, cells were retrieved from the CO_2_ incubator, and their condition was observed. Cells in 80% confluence were selected for harvesting using trypsin-EDTA to detach the cells, followed by centrifugation at 4900 rpm for 5 minutes. The cell count was then determined. Subsequently, the cells were transferred to individual wells, with each well containing 5000−10,000 cells. After filling 12 wells, the cells were resuspended to maintain homogeneity. Two wells were designated as media control and cell control. The cell distribution and morphology were examined under an inverted microscope and documented. The cells were then incubated at 37°C with 5% CO_2_. Once the cells reached 80% confluence, the plate containing the cells was retrieved from the CO_2_ incubator and the cell culture medium was discarded. Next, 100 *μ*l of PBS was added to each well, followed by the removal of the PBS. Serial concentrations of the sample were then added to the respective wells (in triplicate). The cells were incubated in the CO_2_ incubator, with the duration depending on the treatment's effect on the cells. If no cytotoxic effect was observed within 24 hours, further incubation was conducted for an additional duration of 24 hours (total incubation time: 24–48 hours). Towards the end of the incubation period, the cell conditions for each treatment were documented and photographs were taken for all treatment groups except for the media control.

The MTT reagent was prepared by taking 10 *μ*l of the MTT reagent and adding it to each well. The cells were then incubated for 2–4 hours in the CO_2_ incubator until formazan crystals formed. The cell condition was examined using an inverted microscope, and once the formazan crystals were clearly visible, 50 *μ*l of DMSO was added. The plate was then incubated for an additional duration of 15–20 minutes. An ELISA reader was powered on, and the absorbance of each well was measured at a wavelength of 570 nm. After the readings were obtained, the ELISA reader was turned off. Finally, the percentage of viable cells was calculated.

### 2.8. Duration of Decellularization

The duration of the decellularization process was recorded using a stopwatch, starting from the first exposure of the trachea to the decellularizing agent until the completion of the decellularized scaffold.

### 2.9. Statistics

IBM SPSS ver. 19.0 (IBM Corp., Armonk, NY, USA) was used for statistical analysis. Numerical data were presented as the mean and standard deviation. Categorical data were presented as yes (structure observed to be present) and no (structure observed to be absent). For the comparison of variables, analysis of variance (ANOVA) was used as a parametric test for normally distributed data, and if the data distribution was not normal, the Mann–Whitney *U* test was used. Statistical significance was accepted at *P*  <  0.05.

## 3. Results

### 3.1. Pre-Experimental Study

Drawing from the findings of the preliminary study, it was found that the most acellular tracheal group was the one decellularized with H_2_O_2_ for four weeks, while the group that remained cellular was decellularized with 1% SDS for two weeks. Therefore, the decellularization treatments with 0.5% SDS, H_2_O_2_, and a combination of SDS with H_2_O_2_ for two weeks were selected.

Drawing from the findings of the preliminary study, it was found that all tracheas could be sutured using 3.0-size nonabsorbable monofilament thread. Therefore, all treatment groups could proceed to the experimental phase.

### 3.2. MTT Assay

The MTT assay was performed in five different groups: media control, cell control, and samples decellularized with SDS 0.5%, SDS 1%, SDS 1% + H_2_O_2_ 3%, H_2_O_2_ 3%, and control group. Each group consisted of 5 tracheas. The percentage of viable cells is calculated by subtracting the optical density (OD) between the treatment groups and the media control, dividing it by the difference between the OD values of the cell control and the media, and then multiplying the result by 100%. In this study, the treatment group that demonstrated the highest count of viable cells was the one treated with SDS 0.5%. It was followed by the combination of SDS and H_2_O_2_, SDS 1%, and H_2_O_2_ 3%, respectively (Table 2).

Therefore, it can be concluded that the decellularization method using SDS 0.5% is the least toxic, followed by the combination of SDS 1% and H_2_O_2_ 3%. An additional post-hoc analysis was performed, showing that the control group exhibited a significant difference from the rest of the groups and that the combination group is significantly different with the SDS 1% group.

### 3.3. Macroscopic Observation of the Tracheal Scaffold

**Figure 1:**
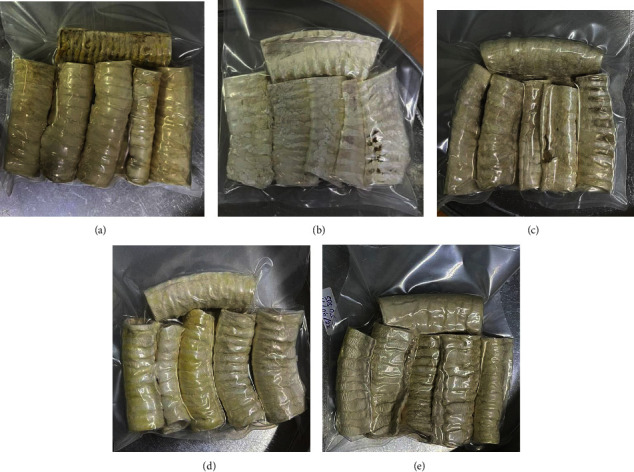
Macroscopic appearance of (a) control group, (b) H_2_O_2_ 3%, (c) combination of SDS 0.5% + H_2_O_2_ 3%, (d) SDS 1%, and (e) SDS 0.5%.

The surface of the natural trachea in the control group displayed a pattern of alternating yellow and white colors, characterized by white cartilage rings and a yellow matrix. Macroscopically, all groups of treatment show the white color of the acellular tracheal matrix, with the whitest color in group H_2_O_2_ 3%, and after that, the combination of SDS 1% and H_2_O_2_ 3%, SDS 1%, and SDS 0.5% (Figure 1).

### 3.4. Histological Analysis

Each treatment group underwent staining with safranin O-fast green (both for GAG staining), hematoxylin and eosin (H&E), and Masson's trichrome (for collagen). Subsequently, the samples were analyzed under a microscope to observe their morphology and the extracellular matrix (ECM).

### 3.5. Hematoxylin and Eosin Staining

**Figure 2:**
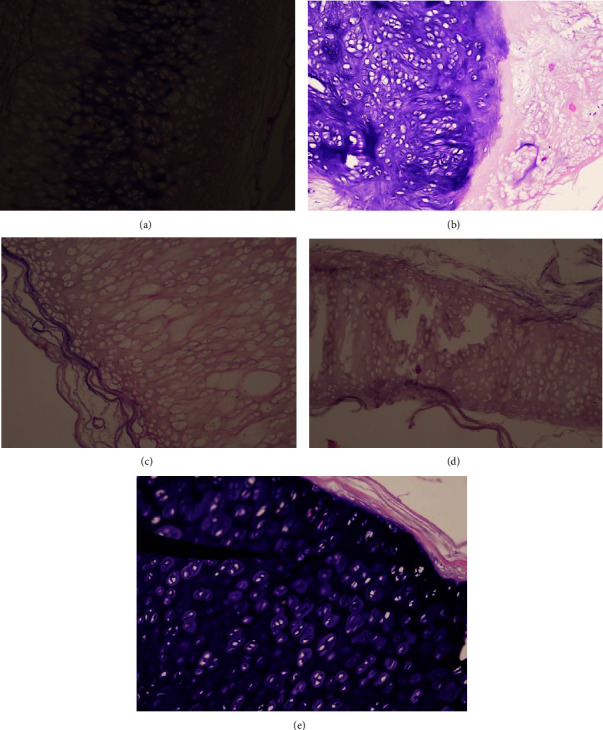
HE staining of (a) SDS 0.5%, (b) SDS 1%, (c) combination of SDS 0.5% + H_2_O_2_ 3%, (d) H_2_O_2_ 3%, and (e) control group.

In this study, the quantitative assessment of the acellularity of the tracheal tissue after the decellularization process was measured using hematoxylin-eosin (HE) staining under a microscope. HE staining was used to observe the structure and composition of the tissue by providing color to the cell nuclei (with hematoxylin) and cytoplasm (with eosin) (Figure 2).

The researchers measured and compared the percentage of the nuclear area between the control group (trachea that had not undergone decellularization) and the treatment group (trachea that had undergone decellularization using various methods or chemical solutions). The results were presented as the mean ± standard deviation in Table 3.

The results of the normality test using the Shapiro–Wilk test for HE (hematoxylin-eosin) data indicated that the data had a normal distribution. The results of the homogeneity test for HE data showed that the data were homogeneous. Based on these normality and homogeneity tests, an ANOVA test was conducted and it was found that *P*  <  0.001, indicating a significant difference among the treatment groups (Table 3). A further post-hoc analysis was conducted, and it showed that the combination group and H_2_O_2_ 3% exhibited a significant difference from the rest of the groups.

### 3.6. Safranin O Staining

Quantitative assessment of safranin staining intensity was performed by comparing the safranin staining content in the treatment group (undergoing decellularization) and the control group. The intensity of safranin staining in the experimental group reflects the remaining amount of proteoglycans in the tracheal tissue after decellularization, while the safranin staining intensity in the control group represents the baseline level of proteoglycans in natural tracheal tissue. Based on the examination of cells under the microscope, they are categorized into values 0 (least GAG component), 1 (minimal GAG component), 2 (sufficient GAG component), and 3 (maximum GAG component).

Based on the normality test, the safranin intensity data are not normal and homogeneous. Based on the Kruskal–Wallis test, a significant difference was found in the results of the safranin O examination among treatment groups (*P*=0.001). A post-hoc analysis using the Mann–Whitney test showed that the control group is significantly different with combination group and H_2_O_2_ 3%. Also, the combination group is significantly different with SDS 1%. Group SDS 1% is also significantly different with the H_2_O_2_ 3% group (Table 4).

The qualitative assessment of glycosaminoglycan (GAG) content in the tracheal tissue after decellularization was measured using safranin O-fast green staining under a microscope.

**Figure 3:**
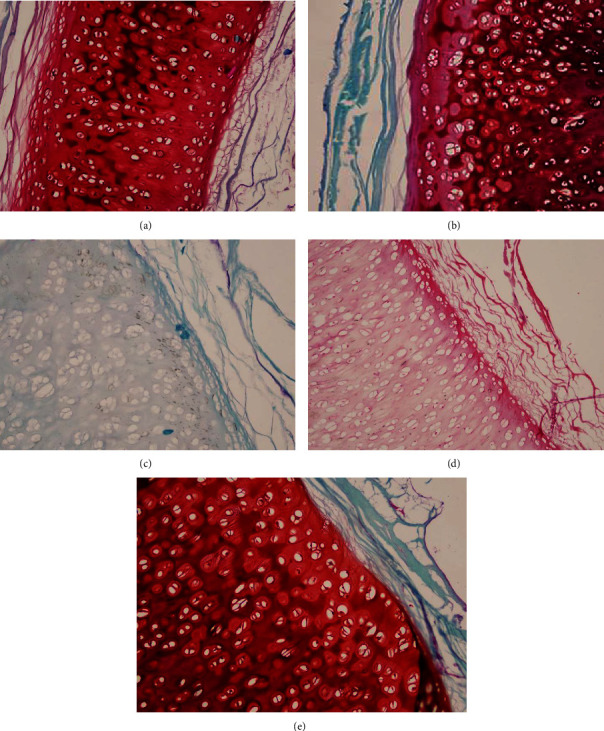
Safranin O staining of (a) SDS 0.5%, (b) SDS 1%, (c) combination of SDS 0.5% + H_2_O_2_ 3%, (d) H_2_O_2_ 3%, and (e) control group.

Safranin O is a dye that specifically stains GAGs, while fast green counterstains the background tissue. This staining technique allows the visualization of GAG-rich regions in the tracheal tissue. Under the microscope, GAGs appear as red or orange-stained areas, while other tissue components are stained in green (Figure 3). This qualitative assessment helps evaluate the effectiveness of the decellularization process in preserving GAG content in the tracheal tissue.

### 3.7. Masson's Trichrome Staining

The qualitative assessment of collagen in the tracheal tissue after the decellularization process was measured using Masson's trichrome staining under a microscope.

**Figure 4:**
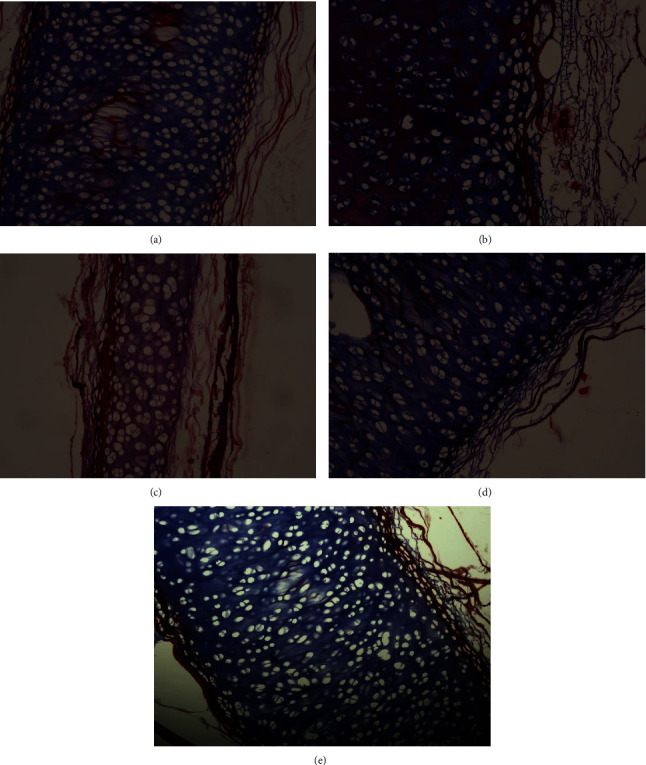
MT staining of (a) SDS 0.5%, (b) SDS 1%, (c) combination of SDS 0.5% + H_2_O_2_ 3%, (d) H_2_O_2_ 3%, and (e) control group.

Masson's trichrome staining is a histological technique that allows the visualization of collagen fibers in tissues. The staining involves multiple steps, including the use of different dyes to selectively stain collagen fibers and other tissue components. Collagen fibers typically appear blue or green in color when stained with Masson's trichrome (Figure 4).

The staining pattern and intensity of collagen fibers can indicate the degree of decellularization and the effectiveness of the process in maintaining the extracellular matrix (ECM) structure. This information is valuable in assessing the quality and suitability of the decellularized tracheal tissue for various applications, such as tissue engineering and transplantation.

By examining Masson's trichrome-stained tracheal tissue sections under a microscope, researchers can evaluate the distribution, abundance, and organization of collagen fibers within the tissue. This qualitative assessment provides insights into the preservation and structural integrity of collagen in the decellularized tracheal tissue. The calculation of Masson's trichrome staining intensity was performed by comparing the intensity in each treatment group with the control group. Based on the examination of cells under the microscope, they are categorized into values 0 (least collagen component), 1 (minimal collagen component), 2 (sufficient collagen component), and 3 (maximum collagen component).

Based on the Kruskal–Wallis test, a significant difference was found in the results of the safranin O examination among treatment groups (*P*=0.001). A post-hoc analysis using the Mann–Whitney test showed that the combination group was significantly different with the rest of the group (Table 5).

### 3.8. Evaluation of Matrix Antigenicity

**Figure 5:**
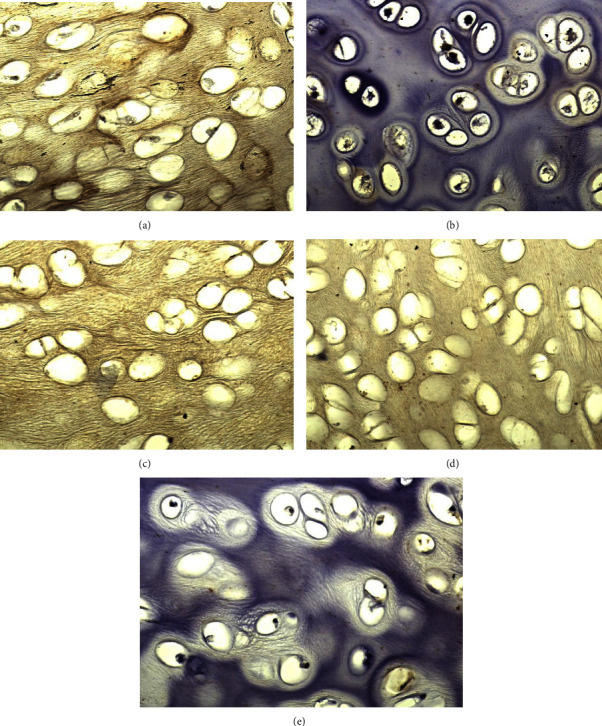
MHC-1 staining of (a) SDS 0.5%, (b) SDS 1%, (c) combination of SDS 0.5% + H_2_O_2_ 3%, (d) H_2_O_2_ 3%, and (e) control group.

The qualitative assessment of the presence or absence of MHC-1 antigen in tracheal tissue after decellularization is measured using anti-MHC-1 staining under a microscope (Figure 5). It is important to evaluate the effectiveness of the decellularization process in removing or reducing immunogenic components from the tracheal tissue. It provides valuable information about the immunocompatibility and potential immune response of the decellularized tissue, helping assess its suitability for further applications.

Anti-MHC-1 staining is an immunohistochemical technique that allows for the visualization and detection of MHC-1 antigens in tissues. This staining method involves the use of specific antibodies that bind to MHC-1 antigens present on the surface of cells. To facilitate their observation under a microscope, the antibodies are tagged with a visible indicator, such as a fluorescent dye or an enzyme, enabling their visualization.

An IRS score was used to calculate the intensity of MHC-1 staining in each sample, providing a quantitative measure of the immunoreactivity. This scoring system takes into account both the percentage of positively stained cells and the staining intensity, and the scoring calculation category is presented as in Table 6, offering a comprehensive evaluation of MHC-1 expression levels across the different tissue samples in Table 7.

The intensity of MHC-1 staining in each sample was quantitatively assessed using an IRS score, providing a measure of immunoreactivity. This scoring system considered both the percentage of positively stained cells and the staining intensity, as depicted in Table 6. The resulting score categories were comprehensively detailed in Table 7, offering a thorough evaluation of MHC-1 expression levels across diverse tissue samples.

### 3.9. Tensile Strength Test

The level of biomechanics after organ decellularization treatment is used as an indicator to assess the durability of the scaffold after the treatment. In this study, the biomechanical aspect is calculated by measuring the average tensile strength of the samples in each treatment group and comparing them with the control group, which represents the standard or baseline value (Table 7).

The tensile strength test is conducted to assess the durability and mechanical stability of the scaffold after decellularization treatment, utilizing the Mini Bionix II Universal Test Device and applying tension using the ESIT Load Cell (SPA-10 kg, output 2.0 mV/V). This test provides valuable insights into the effectiveness of the treatment in preserving the desired biomechanical properties of the scaffold.

The biomechanical status after organ decellularization treatment serves as an indicator to evaluate the scaffold's resistance following the treatment. In this study, biomechanical aspects were computed by measuring the average tensile strength of samples in each treatment group and comparing them with the control group, representing the standard or baseline values.

The results of the normality test using the Shapiro–Wilk test for the tensile strength test indicate that the data have a normal distribution. The homogeneity test for the tensile strength test shows that the data are homogeneous. Based on these normality and homogeneity tests, an ANOVA test was conducted, revealing a significant difference among the treatment groups with a *P* value of 0.49 (*P*  <  0.05). A further post-hoc analysis was conducted, and it showed that the combination group and H_2_O_2_ 3% exhibited a significant difference from the rest of the groups (Table 8).

This analysis helps evaluate the durability and mechanical stability of the scaffold after the decellularization treatment. It provides valuable insights into the effectiveness of the treatment in maintaining the desired biomechanical properties of the scaffold for its intended applications.

## 4. Discussion

The objective of this study is to identify the decellularization technique that can provide scaffolds with minimal cellular nucleus concentration, sufficient glycosaminoglycan concentration, high tensile strength and elasticity, and a faster decellularization phase.

### 4.1. Pra-Experimental Study

Currently, most tracheal scaffold decellularization is performed using detergent-enzymatic methods (DEM), which remove tracheal antigens while preserving the three-dimensional structure and extracellular matrix (ECM) of the trachea, providing a compatible and supportive environment for grafting [15, 21–24]. Wang et al., using the detergent-enzymatic method (DEM) with increased DNase concentration on rabbit trachea, showed shorter decellularization time (2 cycles/4 days) while eliminating rabbit antigens and maintaining the intact ECM structure [23].

In this study, a chemical-only decellularization method was used, similar to the study by Dimou et al., where rat trachea was decellularized in SDS, CHAPS, and FBS solution for three cycles (approximately 12 days), demonstrating effective tracheal decellularization with intact ECM matrix and no toxicity [25].

So far, there have been no studies using goat trachea as a scaffold in tracheal tissue engineering. Therefore, the researchers conducted a pre-experimental study to determine the appropriate duration and concentration of chemical solutions for effective decellularization of goat trachea. Based on the pre-experimental study, it was found that trachea decellularized with H_2_O_2_ solution for four weeks resulted in the most cellular removal, while SDS 1% solution for one week still showed cellular presence. Therefore, several treatment groups were selected using H_2_O_2_ 3% solution, SDS 1% solution, SDS 0.5% solution, and a combination of SDS 0.5% and H_2_O_2_, which were processed for four weeks.

### 4.2. MTT Assay

In this study, cytotoxicity testing was quantitatively assessed using the MTT assay, which involves measuring the optical density to provide a quantitative evaluation of cellular toxicity. The MTT assay is typically performed after a few hours of incubating cells with MTT. The resulting water-insoluble formazan is solubilized using a solvent like DMSO. The homogenized MTT-formazan solution's optical density (OD) is then measured at around 570 nm using a microplate reader, which indicates the formazan concentration and intracellular reduction of MTT. For nearly four decades, the MTT assay has been widely used to assess cell proliferation/viability, drug cytotoxicity, and cellular mitochondrial/metabolic activity [26].

The results showed the lowest toxicity in the group using SDS 0.5%, followed by SDS 0.5% + H_2_O_2_, SDS 1%, and H_2_O_2_, respectively. These results are consistent with other studies that utilized different chemical decellularization methods [21, 23, 24, 27], which also demonstrated low toxicity in decellularized trachea. A study conducted by Wang et al. utilized modified DEM treatment for tracheal decellularization in rabbits, demonstrating minimal toxicity as measured by optical density, similar to the findings in our study. These consistent findings across various studies underscore the robustness of the decellularization methods employed in minimizing toxicity [21].

### 4.3. Effectiveness of the Decellularization Method

Histological analysis was performed to assess the effectiveness of the decellularization method. Specimens stained with hematoxylin and eosin (H&E) were used to evaluate the cellular nuclei, while the ECM matrix was analyzed using safranin O-fast green staining (for glycosaminoglycan/GAG content) and Masson's trichrome staining (for fibrin and collagen structure within the ECM). In this study, it was found that the decellularization method with the least amount of cellular nuclei was achieved using a 3% H_2_O_2_ solution. Meanwhile, the solutions that preserved the highest levels of glycosaminoglycan content and maintained the fibrin and collagen structure were the 0.5% SDS solution and the 3% H_2_O_2_ solution.

After the decellularization process, the tracheal matrix exhibits commendable biocompatibility and maintains the integrity of the tracheal extracellular matrix (ECM) [12, 21]. The persistence of essential proteins such as collagen and glycosaminoglycans (GAGs) within the ECM plays a crucial role in defining the mechanical characteristics of the tracheal matrix [28, 29]. Notably, our observations indicate that the collagen fibers in the experimental group remained largely unchanged following the decellularization process. This study is similar to the research conducted by Wang et al., who also used a combination of detergent and enzymatic solutions as a decellularization method for rabbit trachea, resulting in an intact ECM structure with minimal cellular nuclei [23]. Similarly, in the study by Dimou et al., which used a chemical decellularization method similar to this research, low DNA content and preserved glycosaminoglycan and ECM matrix levels were observed [25].

### 4.4. Analysis of Antigenicity on ECM

In addition to histological evaluation using various staining techniques, the immunohistochemical examination is also necessary to detect residual markers of immunological factors on the scaffold, in this case, anti-MHC-1. The presence of these antigens can increase recipient rejection reactions [30, 31].

The primary challenge in the success of tracheal replacement has been the immunological rejection of tracheal grafts, with its immunogenicity primarily originating from the epithelial and mucosal lamina propria [28, 29, 32]. The decellularization technology efficiently dissolves epithelial and mucosal cells in the natural trachea using detergents and enzymes. This process removes the immunogenicity of the matrix, eliminating the necessity for immunosuppressive agents to reduce the risk of rejection after surgery [31, 33].

A study by Sun et al. showed the disappearance of MHC-II antigen in rabbit trachea after undergoing a 7-cycle decellularization method [34]. Another study by Wang et al., with an increased concentration of DNase, only required two cycles to remove antigens from both MHC-1 and MHC-II [23]. Both studies used a detergent-enzymatic decellularization method.

From the examination of MHC-1 antigens, it was found that the groups treated with the decellularization method using 3% H_2_O_2_ and 0.5% SDS + 3% H_2_O_2_ yielded the lowest results successively. However, in the treatment groups with 0.5% SDS and 1% SDS, higher results were obtained compared to the control group. This may be attributed to the extravitally occurring processes in the goat trachea from the time of acquisition until the decellularization treatment. It may also be due to the activity of still-active cells in the 0.5% and 1% SDS treatment groups after the treatment. In this study, it is challenging to ensure that all treatment groups have the same initial cell count, considering the use of biological samples such as goat trachea. Further in-depth research on MHC-1 antigens is needed to obtain a more comprehensive understanding. These findings are specific to our study and contribute to the current understanding of decellularization effects on MHC-1 antigens in goat trachea.

### 4.5. Tensile Strength Test

This study revealed a variation in tensile strength between the control group and the decellularized groups. The group with the highest stress resistance was group C (SDS 0.5% + H_2_O_2_), followed by group A (SDS 0.5%), group B (SDS 1%), and group D (H_2_O_2_) in that order. This finding is similar to the study conducted by Wang et al., which also reported a difference in tensile strength between the control group and the decellularized group [35]. This difference is attributed to the potential cell-damaging effects of the chemicals used. However, various studies, including one conducted by Batioglu-Karaaltin et al., have suggested that the tensile properties of the tracheal matrix after decellularization closely resemble those of native tracheas. The study found no significant difference in tensile strength between the control group and the decellularized group [20, 21, 36].

## 5. Conclusions

In summary, the combination of 1% SDS and 3% H_2_O_2_ for the decellularization of goat trachea can produce a tracheal scaffold with minimal cell nuclei, low toxicity, preserved extracellular matrix, and high tensile strength and elasticity. This study provides data that support the development of decellularization techniques for creating a well-functioning tracheal scaffold to address various severe respiratory diseases.

As a suggestion for future research, experiments could be conducted with variations in SDS concentration and a lower concentration of 3% hydrogen peroxide to determine the most optimal combination in producing tracheal scaffolds with the best quality. Additionally, it is advisable to measure the initial cell count before applying decellularization treatment to each group as a baseline to ensure a consistent and comparative cell count throughout the entire study. By conducting more in-depth and varied research, it is hoped that the results will provide better insights into the potential use of these tracheal scaffolds in medical and regenerative applications.

## Figures and Tables

**Table 1:** The formula for the sample size in each ANOVA design.

ANOVA design	Application	Minimum number of n per group	Maximum number of n per group
One-way ANOVA	Comparison between groups	10/*k* + 1	20/*k* + 1
One within-factor, ANOVA with repeated measures	One group, repeated measurements	10/(*r* − 1) + 1^a,b^	20/(*r* − 1) + 1^a,b^
One between-factor, one within-factor, ANOVA with repeated measures	Comparison between groups, repeated measurements	10/*kr* + 1^b^	20/*kr* + 1^b^

*k* = number of groups, *n* = number of subjects per group, *N* = total number of subjects, *r* = number of repeated measurements. ^a^*n* = *N*, because only one group is involved, ^b^*n* must be multiplied by *r* if there are sacrificed animals in each measurement.

**Table 2:** The percentage of viable cells after being decellularized for each group of treatments.

Treatment group	Number of samples (*n*)	Percentage of cell retaining nuclei (%) (mean ± SD)	ANOVA sig. (*P* value)
Control	5	36.99 ± 10.95^b^	0.001
SDS 0.5%	5	78.87 ± 23.8^a^	
SDS 1%	5	66.66 ± 12.04^a,^^*∗*^	
SDS 0.5% + H_2_O_2_ 3%	5	76.36 ± 14.83^a,^^*∗*^	
H_2_O_2_ 3%	5	51.25 ± 15.75^a^	

^a-b^, Within a row, means without a common superscript differ (*P*  <  0.05) and ^*∗*^common superscripts show a significant difference (*P*  <  0.05).

**Table 3:** The percentage of cells retaining nuclei for each group of treatments.

Treatment group	Number of samples (*n*)	Percentage of cell retaining nuclei (%) (mean ± SD)	ANOVA sig. (*P* value)
Control	5	52 ± 10.95^a^	0.001
SDS 0.5%	5	51 ± 23.8^a^	
SDS 1%	5	53 ± 12.04^a^	
SDS 0.5% + H_2_O_2_ 3%	5	18 ± 14.83^b^	
H_2_O_2_ 3%	5	15.4 ± 15.75^b^	

^a-b^, Within a row, means without a common superscript differ (*P*  < 0.05).

**Table 4:** The percentage of safranin intensity for each group of treatments.

Treatment group	Number of samples (*n*)	Intensity of safranin staining (%) med (min-max)	Kruskal–Wallis sig. (*P* value)
Control	5	4.00 (4.00-4.00)^a^	0.001
SDS 0.5%	5	3.00 (0.00–4.00)	
SDS 1%	5	4.00 (4.00-4.00)^b^	
SDS 0.5% + H_2_O_2_ 3%	5	1.00 (0.00-1.00)^a^	
H_2_O_2_ 3%	5	3.00 (2.00–4.00)^ab^	

^a-b^, Within a row, medians with a common superscript differ (*P*  <  0.05).

**Table 5:** The degree of collagen staining density using Masson's trichrome in each treatment group.

Treatment group	Number of samples (*n*)	Intensity of Masson's trichrome staining (%) med (min-max)	Kruskal–Wallis sig. (*P* value)
Control	5	3.00 (2.00-3.00)^a^	0.001
SDS 0.5%	5	2.00 (2.00-3.00)^a^	
SDS 1%	5	2.00 (2.00-2.00)^a^	
SDS 0.5% + H_2_O_2_ 3%	5	0.00 (0.00–2.00)^b^	
H_2_O_2_ 3%	5	3.00 (0.00–3.00)^a^	

^a-b^, Within a row, median without a common superscript differ (*P*  <  0.05).

**Table 6:** The calculation and interpretation of MHC-1 intensity using the IRS score.

Proportion	Staining intensity	Proportion score (PS)/intensity score
0%	Negative	0
<10%	Weak	1
10%–50%	Intermediate	2
51%–80%	Strong	3
>80%	—	4
*Interpretation (PS∗IS)*		
0-1	Negative	
2-3	Positive: weak	
4–8	Positive: moderate	
9–12	Positive: strong	

**Table 7:** The intensity of MHC-1 measured in IRS score for each treatment group.

Treatment group	Number of samples (*n*)	IRS score med (min-max)	Kruskal–Wallis sig. (*P* value)
Control	5	2.50 (0.00–3.50)	0.001
SDS 0.5%	5	3.00 (0.00–7.50)	
SDS 1%	5	6.00 (2.25–7.00)^ab^	
SDS 0.5% + H_2_O_2_ 3%	5	0.00 (0.00–2.00)^a^	
H_2_O_2_ 3%	5	0.00 (0.00–2.50)^b^	

^a-b^, Within a row, medians with a common superscript differ (*P*  <  0.05).

**Table 8:** The average tensile strength values (MPa) in each treatment group.

Treatment group	Number of samples (*n*)	Percentage of cell retaining nuclei (%) (mean ± SD)	ANOVA sig. (*P* value)
Control	5	2.29 ± 0.66^a^	0.049
SDS 0.5%	5	2.19 ± 0.33^a^	
SDS 1%	5	2.05 ± 0.27^a^	
SDS 0.5% + H_2_O_2_ 3%	5	3.13 ± 1.01^b^	
H_2_O_2_ 3%	5	1.99 ± 0.4^a^	

^a-b^, Within a row, means without a common superscript differ (*P*  <  0.05).

## Data Availability

All data generated or analyzed in this study are included within the published article and can be obtained from the respective authors upon reasonable request.
